# Preparation and properties of rigid polyurethane foams added with graphene oxide-hollow glass microspheres hybrid

**DOI:** 10.1080/15685551.2021.1954340

**Published:** 2021-07-14

**Authors:** Dong Liu, Longqing Zou, Qianqian Chang, Tianyuan Xiao

**Affiliations:** a School of Mechanical Science and Engineering, Northeast Petroleum University, Daqing, China; b School of Mechanical and Electronic Engineering, Qiqihar University, Qiqihar, China

**Keywords:** Rigid polyurethane foam, graphene oxide, hollow glass microspheres, hybrid, foam-filled tubes, energy absorption

## Abstract

Rigid polyurethane foam (RPUF) as a filling material that can enhance the crashworthiness of thin-walled tubes. GO-HGMS hybrid was prepared by solution blending of graphene oxide (GO) and hollow glass microspheres (HGMS). The effect of the composite on the compression properties of RPUF was investigated. The GO-HGMS hybrid was characterized by fourier transform infrared spectroscopy (FTIR), x-ray diffraction(XRD), and scanning electron microscopy (SEM). The compression test and microstructure results show that the best compression performance and the largest apparent density of the composite foam were obtained when the hybrid content was 4 wt %. In addition, the compression test results of empty tubes (ET) and foam-filled tubes (FFT) under lateral load indicate that the combination of lightweight foamed material and thin-walled tube improves the stability of thin-walled tube deformation and the ability of the structure to resist deformation. GO-HGMS/RPUF as the filling material of thin-walled tube structure greatly improves the bearing capacity and energy absorption level of ET.

## 1. Introduction

Improving the crashworthiness of a structure by designing different forms of energy absorbers is an important subject in engineering design. Thin-walled tubes are easy to process and manufacture and have high stiffness/weight ratio and thus have been widely used as energy absorption devices in transportation, aviation, and protection engineering [1–4]. Studies have investigated the material, geometric characteristics, and loading mode of circular tube structure [5–8]. Thin-walled tubes are weak to bear lateral load; composite foam-filled tubes formed by the combination of porous energy absorbing materials and thin-walled metal tubes has obvious advantages to meet the requirements of the engineering application field for structural load bearing and energy absorption characteristics and achieve the design requirements of lightness and high strength. Filling materials are the key to determine the structural properties of filled tubes. Common lightweight porous materials include foam metals, polymer foams, and porous biomaterials [9–16].

Rigid polyurethane foam (RPUF) plays an important role in polymer foamed materials and has excellent physical and mechanical properties, such as low density, low thermal conductivity, and good shock absorption properties. Traditional particles (calcium carbonate, aluminum hydroxide, glass microspheres) and nanoparticles (graphene, carbon nanotubes, nano-silica) are used as reinforcing materials to improve the mechanical properties of rigid polyurethane foam [17–22]. Compared with traditional particles, the amount of nanoparticles filler is less, which has little effect on the foaming process of polyurethane foam. In general, the reinforcing effect of filler is closely related to its dispersion in the matrix. Graphene oxide (GO), as a typical nanomaterial, has the characteristics of small size effect, large specific surface area, and high strength and can be mixed with polymer materials to prepare nanocomposites with excellent mechanical properties [23–25]. However, dispersing GO in the raw materials of polyurethane is difficult because it easily causes agglomeration and affects the properties of foam composites. Hollow glass microspheres (HGMS) have spherical structure and smooth surface as well as high compressive strength, good fluidity, and stability; as such, HGMS can be effectively dispersed by mechanical stirring. However, when HGMS is used as a filler, it often needs to be added in large amounts, and the increase in the strength of the foam is limited [26].

GO and HGMS have different structures and properties and have their own advantages in practical application. If they are used at the same time, they have certain potential value. However, limited studies have been conducted on RPUF-reinforced fillers. The surface of GO is rich in organic functional groups (─OH, ─COOH, ─C═O), which can be combined with various inorganic particles through chemical bonds [25]. Inorganic particles can increase the distance between the layers of GO and form a kind of hybrid filler with a three-dimensional structure, which improves the dispersion of the particles in the polymer materials. Li et al. [27] prepared GO and fly ash cenospheres (FACs) hybrid through solution blending. Their results show that the GO-FAC hybrid filler has a significant effect on improving the tensile and wear resistance of epoxy resin composites; when the content is 0.5 wt%, both properties are optimal. Ramezanzadeh et al. [28], Bouibed et al. [29], and Pourhashem et al. [30] prepared GO-SiO_2_ hybrids and filled them into epoxy coating; they reported that the introduction of GO-SiO_2_ hybrids into the epoxy coating enhances the corrosion resistance and thermal stability.

The main purpose of this study is to improve the compressive mechanical properties of RPUF, then improve the energy absorption capacity of the foam-filled tubes by using GO-HGMS hybrid as filler. This paper describes the principle and morphological characteristics of the preparation of GO-HGMS hybrid and compares the influence of different contents of GO-HGMS on the compression properties of foam materials. Moreover, the lateral compression deformation behavior of empty tubes (ET) and foam-filled tubes (FFT) are studied, and a load-displacement curve is established. The deformation failure mode, compressive bearing mechanical properties, and energy absorption characteristics of the structure are analyzed.

## 2. Materials and methods

### 2.1. Materials

GO-HGMS was prepared using the following materials: silane coupling agents (KH550, Dongguan Kangjin Material Co., Guangdong, China); dimethyl-formamide (DMF) (analytical reagent) and absolute ethyl alcohol (analytical reagent) (Jintongletai Chemical Co., Beijing, China); graphene oxide (layer thickness = 0.6–1 nm, Hengqiu Technology Co., Suzhou, China); and HGMS (S60HS, density = 0.60 g/cm^3^, 3 M Co., Minnesota, America).

The thin-walled tube material used was 6061 aluminum alloy with an outer diameter of 46 mm, a wall thickness of 1 mm, and a length of 40 mm. Internal filled polyurethane foam was obtained by free foaming. Polyurethane A and B materials were purchased from Jining Huakai Resin Co., Ltd.(Shandong, China).

### 2.2. Preparation of GO-HGMS

**Figure 1.**
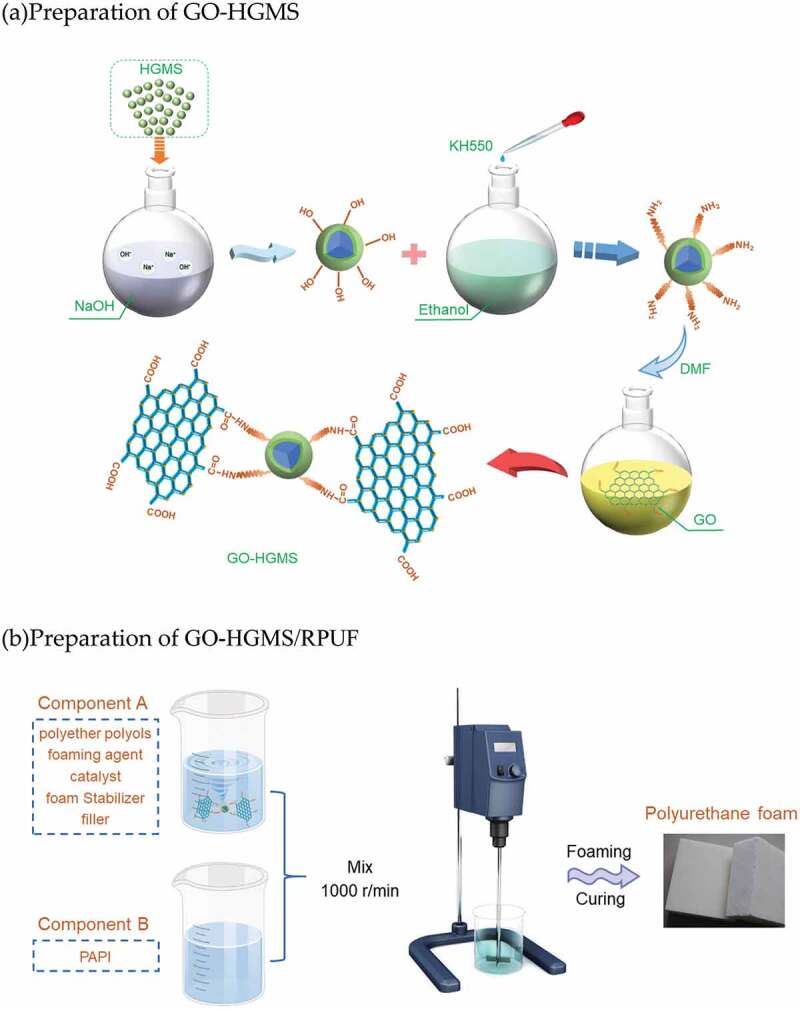
Schematic diagram of GO-HGMS hybrid preparation(a) as well as the resulting polyurethane foam (b)

The principle of GO-HGMS hybrid preparation is shown in Figure 1(a) [27]. HGMS was added to sodium hydroxide solution with a concentration of 0.3 mol/L. The mixture was stirred in a Oil bath at 80 °C for 2 h for hydroxylation treatment. About 1 g of KH550 was added to 300 ml of ethanol-water (1:3, v/v) mixed solution, and dried HGMS was added. The mixture was refluxed and stirred at 80 °C for 3 h. In brief, 1 g of GO and 2 g of modified HGMS were added into 100 ml of DMF solvent and reacted in a water bath for 2 h at 90 °C. The stirring reaction speed was set at 50 rpm. Finally, the mixture was filtered under reduced pressure and cleaned with ethanol solution. The mixture was placed in a vacuum drying oven and dried at 70 °C for 12 h to obtain the powder sample.

### 2.3. Preparation of GO-HGMS/RPUF

The fabrication process of polyurethane composite foam is shown in Figure 1(b). About 30 g of polyurethane A material was weighed and added with the corresponding mass fraction of GO-HGMS hybrid (0, 1, 2, 3, 4, 5, 6,and 7 wt %). The mixture was stirred for 5 min and then added with 30 g of polyurethane B. After mixing at 800 r/min for 10 to 30 seconds, it was injected into the mold to freely foam at room temperature. The product was placed at room temperature for 24 h and cut into a standard sample with a size of 50 mm×50 mm×50 mm. The mixture of A and B materials was injected into the aluminum tube according to similar method, and the FFT was cut and polished. Quasi-static lateral compression test was conducted on a 100 KN CMT5105 electronic universal testing machine at a constant of 2 mm/min.

## 3. Results and discussion

### 3.1. Characterization of GO-HGMS

**Figure 2.**
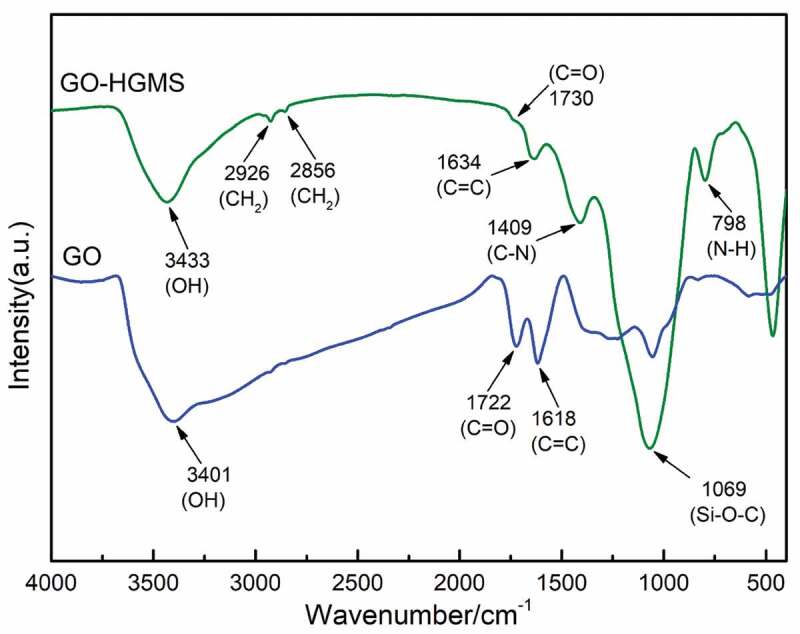
FTIR spectra of GO and GO-HGMS

FTIR characterization tests were performed for GO and GO-HGMS solid powders (Figure 2). The stretching vibration characteristic peak of ─OH at 3401 cm^−1^ in GO-HGMS is obviously weaker than that in GO due to the reaction of GO with the intermediate product of reactants. In the GO-HGMS spectrum of the product, peaks appear at 2856 and 2926 cm^−1^, which are the symmetric and antisymmetric absorption peaks of ─CH_2_. In the FTIR spectrum of GO, peaks at 1722 and 1618 cm^−1^ indicate the presence of C═O and C═C functional groups, respectively. Compared with the FTIR spectra of GO-HGMS, the C-O-C absorption peak at 1226 cm^−1^ disappears, and new absorption peaks appear at 1409, 1069, and 798 cm^−1^, which are attributed to C-N, Si-O-C, and N-H absorption, respectively [29]. In conclusion, in the reaction, the intermediate structure of ─NH_2_ obtained by HGMS and KH550 reacted with the ─COOH of the reactant GO to produce a new compound, namely, GO-HGMS.

**Figure 3.**
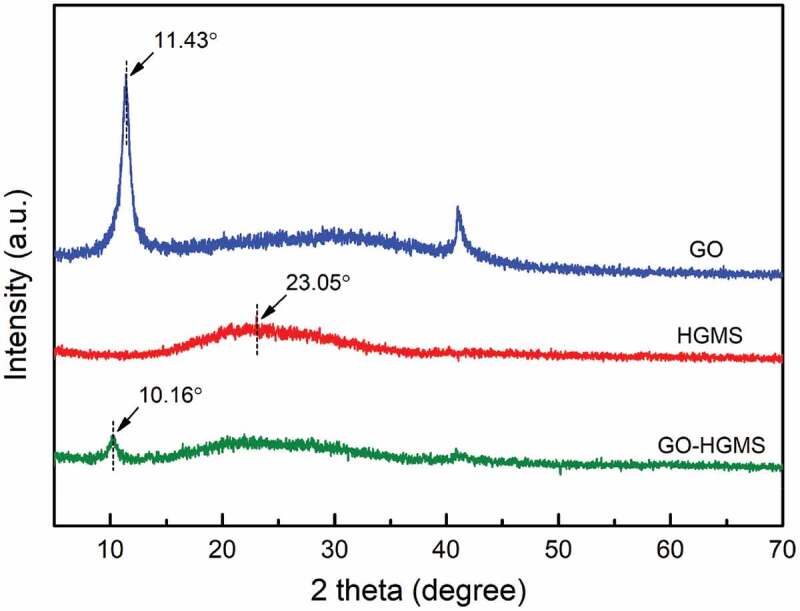
XRD spectra of neat GO, HGMS and GO-HGMS hybrid

The composition and structure of GO-HGMS hybrid were analyzed by XRD, and compared with pure GO and HGMS, as shown in Figure 3. The neat GO has an obvious characteristic diffraction peak at 2θ = 11.43º, indicating that the lamellar spacing was approximately 0.774 nm. The HGMS XRD spectra show that a broad diffraction peak appears near approximately 2θ = 23.05º, corresponding to the typical diffraction peak representing the amorphous structure of the HGMS. The XRD pattern of the GO-HGMS hybrid showed that the two diffraction peaks of GO and HGMS appeared. The weak diffraction peaks appeared at about 2θ = 10.16º with a lamellar spacing of 0.870 nm. Compared with GO, the diffraction peak moves to the left and HGMS is inserted into the interlayer of GO, which makes the interlayer spacing increase and forms a complete and ordered structure. Meanwhile, the characteristic diffraction peak of GO is also significantly weakened due to the deposition of HGMS on the GO surface.

**Figure 4.**
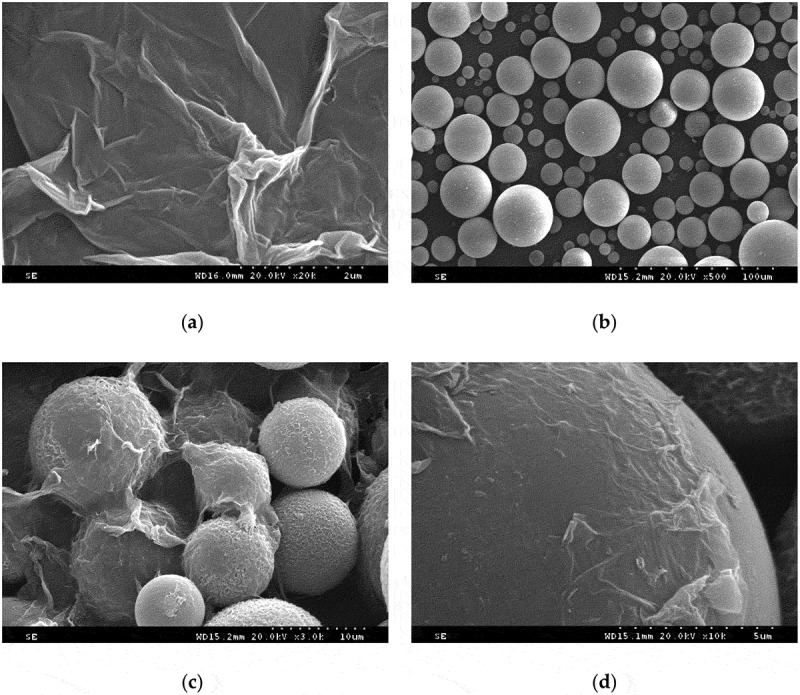
SEM images of (a) GO, (b) HGMS, (c) GO-HGMS, and (d) local of GO-HGMS

The SEM images of GO, HGMS and GO-HGMS are shown in Figure 4. Figure 4(a) shows that GO has a very thin sheet structure, and the fold of the surface increases the specific surface area, which is prone to agglomeration. Figure 4(b) shows that the surface of the HGMS spherical structure is smooth and has good fluidity and dispersion. With GO modification, the HGMS surface is covered with thin and wrinkled GO layers (Figure 4(c,d)). Loading of HGMS increases the distance between lamellae on the GO surface and drives the GO to achieve good dispersion in the polyurethane foam material.

### 3.2. Compressive properties of polyurethane composite foam

**Figure 5.**
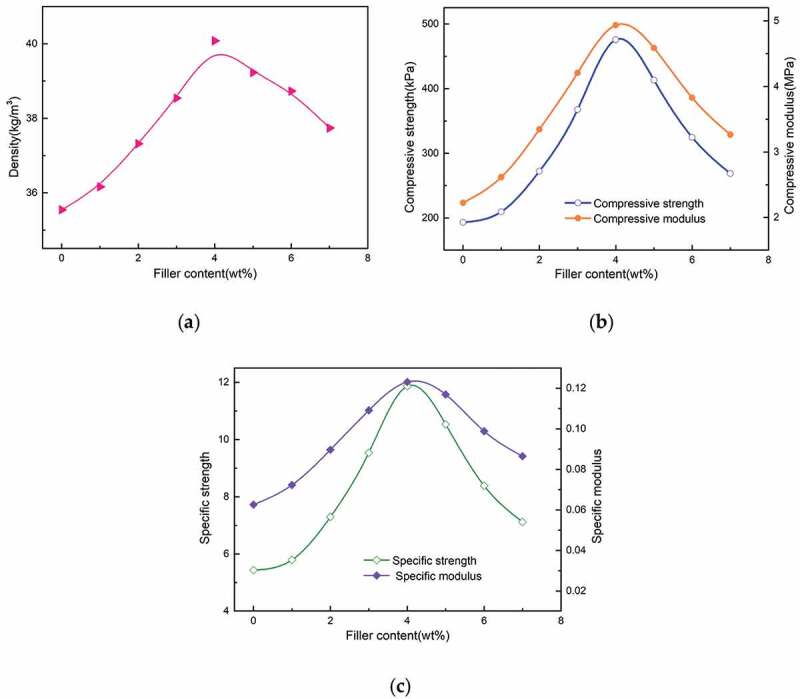
The apparent density (a),compressive strength and modulus (b) and specific strength and modulus (c) of samples with different contents

As shown in Figure 5(a), the apparent density of the foam increases continuously with the increase of GO-HGMS content, and the maximum value appears in the filler content of 4 wt% (40.08 kg/m3). When more than 4%, the apparent density of foam decreases with the increase of filler content. Apparent density were reduced when the filler content was more than 4 wt %. GO-HGMS hybrid combines the advantages of GO and HGMS, which greatly increases the compressive strength and compression modulus of the composite foam. As shown in Figure 5(b), 4.0 wt% GO-HGMS/RPUF shows the best compression performance, and the maximum compression strength is 475.6 kPa, which is 146.17% higher than pure foam (193.2 kPa). The maximum compression modulus is 4.935 MPa, which is 121.90% higher than that of pure foam (2.224 kPa). Similar trends of change appeared for the specific strength and specific modulus as shown in Figure Figure 5(c). However, at high filler content, the GO agglomeration in the matrix becomes a stress concentration point, which reduces the enhancement effect on the foam matrix.

**Figure 6.**
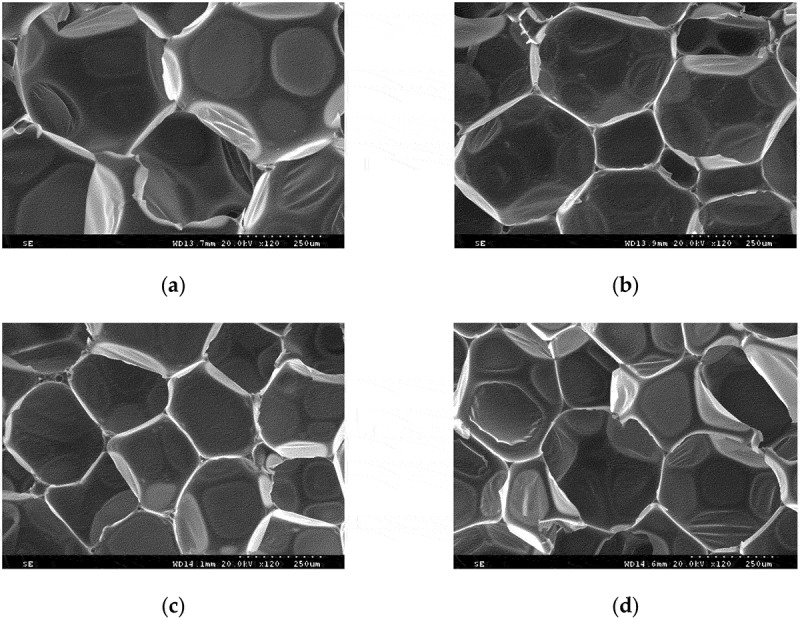
SEM images of (a) pure and GO-HGMS with (b) 1 wt%, (c) 4 wt%, and (d) 7 wt%

The microstructure of the prepared PRPUF and GO-HGMS/RPUF with different contents is shown in Figure 6. The cell shape of the PRPUF is hexagon. The addition of low content of the filler improves the structure of the cells and reduces the diameter of the cells. However, the addition of fillers makes the uniformity of the cells worse, and cells with larger pore diameter and smaller pores exist at the same time. When the filler content is 4 wt%, the smallest diameter of the cell is obtained, and the structure is relatively uniform. The addition of fillers enhances the strength and hardness of the pore wall and allows the formation of dense bubbles, thereby improving the compressive strength of RPUF. When increasing the content of GO-HGMS, the increase in the filler content may lead to some agglomeration, which interferes with the foaming reaction of polyurethane and hinders the improvement of the cell wall. The cell diameter increases again, and the compressive mechanical properties begin to decrease.

### 3.3. Lateral compression of foam-filled tubes

**Figure 7.**
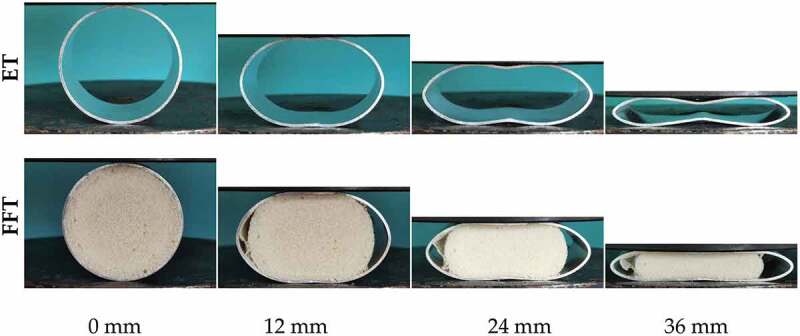
Deformation mode of ET and pure/FFT under lateral compressive force

Figure 7 shows the deformation modes of ET and FFT under lateral compression. With the increase of compression displacement, the internal and external wall of ET is subjected to uneven force. The outer layer of the tube wall is compressed, and the inner layer is pulled, which leads to the collapse deformation of the tube to the inner concave curve. The contact area gradually transitions from the middle to the arc region on both sides, showing an axisymmetric deformation mode. For FFT, the progressive collapse characteristics of the upper and lower ends are not obvious, no obvious inward collapse is formed, and the contact plane between the indenter and the tube expands laterally due to the internal effect of the foam filler. The combination of the thin-walled tube and RPUF makes the deformation mode of the structure more stable and enhances the ability of the structure to resist deformation [31].

**Figure 8.**
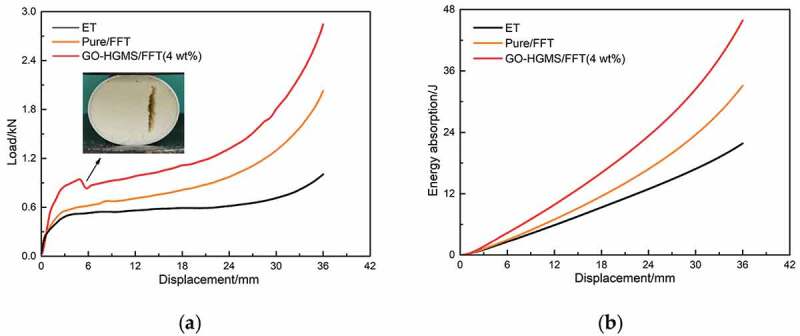
Load-displacement curves (a) and energy absorption capacity (b) of ET and FFT

The load displacement curve and energy absorption of ET and FFT are shown in Figure 8. At the 4 mm position, the load-displacement curve of GO-HGMS/FFT has a sudden change because the composite foam matrix is broken at this time. After adding inorganic filler to RPUF, the compatibility between the matrix and the filler becomes worse with increasing filler quality, and the material undergoes brittle fracture. The results show that the maximum compression force of ET is 1.02 kN, that of Pure/FFT is 2.03 kN, and that of GO-HGMS/FFT is 2.84 kN, which is 178.43% higher than that of ET. Compared with the average compression force of the three kinds of tubes (ET, 0.61 kN; Pure/FFT, 0.92 kN; and GO-HGMS/FFT, 1.27 kN), GO-HGMS/FFT is 108.93% higher than ET. Compared with that of the ET, the values of Pure/FFT and GO-HGMS/FFT increase by 51.69% and 110.07% respectively.

The carrying capacity and energy absorption capacity of FFT have been greatly improved compared with those of ET. After combining a lightweight foam material with a thin-walled tube, the foam material has an inhibitory effect on the collapse and bending deformation of the tube wall during compression. The contact area between the indenter and the tube increases, and the number of crystal particles participating in deformation and energy absorption increases [15]. The mechanical properties of the foam material have a significant impact on the structural performance of the filled tube. The GO-HGMS/RPUF has greatly improved the load-bearing capacity and energy absorption capacity of ET and has excellent performance as a filling material for circular tube structure.

## 4. Conclusions

This study prepared GO-HGMS hybrid by solution blending and characterized it by FTIR, XRD and SEM analyses. The results confirmed the successful loading of HGMS on the GO surface. The GO-HGMS/RPUF composite foam was synthesized by free foaming. When the content of GO-HGMS increases, the cell diameter first decreases and then increases, and the mechanical properties of the syntactic foam first increase and then decrease. The 4 wt% GO-HGMS/RPUF composite foam has the largest apparent density, the smallest and more uniform cell structure, and its compression strength is 146.17% higher than pure foam. The GO-HGMS/RPUF composite foam can enhance the ability of the FFT structure to resist deformation in stable deformation mode, thereby improving the bearing capacity and energy absorption level of ET. The average compression force and total energy absorption of the GO-HGMS/FFT structure are increased by 108.93% and 110.07% compared with those of ET.
